# m^6^A RNA Methylation Decreases Atherosclerotic Vulnerable Plaque Through Inducing T Cells

**DOI:** 10.21470/1678-9741-2021-0039

**Published:** 2023

**Authors:** Chunmei Qi, Haoran Li, Yongshu Yu, Ji Hao, Hao Zhang, Lele Wang, Jingjing Jin, Qiang Zhou, Ya Hu, Chengmeng Zhang, Qingdui Zhang

**Affiliations:** 1 Department of Cardiology, Second Affiliated Hospital of Xuzhou Medical College, Xuzhou, Jiangsu, People’s Republic of China.; 2 Department of Cardiology, Xuzhou Medical University, Xuzhou, Jiangsu, People’s Republic of China.; 3 Department of Cardiology, The Second Hospital of Xuzhou Mining Group, Xuzhou, Jiangsu, People’s Republic of China.

**Keywords:** Obesity, RNA, Small Interfering, Plaque, Atherosclerotic, IL & protein, human, Interleukin-7, Alpha-Ketoglutarate-Dependent Dioxygenase FTO

## Abstract

**Introduction:**

Knockdown of fat mass and obesity-associated gene (FTO) can induce N6-methyladenosine (m^6^A) ribonucleic acid (RNA) methylation. The objective of this study was to explore the effect of m^6^A RNA methylation on atherosclerotic vulnerable plaque by FTO knockdown.

**Methods:**

A total of 50 New Zealand white rabbits were randomly divided into pure high-fat group, sham operation group, vulnerable plaque group, empty load group, and FTO knockdown group (10 rabbits/group).

**Results:**

Flow cytometry showed that helper T (Th) cells in the FTO knockdown group accounted for a significantly higher proportion of lymphocytes than in the vulnerable plaque group and empty load group (*P*<0.05). Th cells were screened by cell flow. The level of m^6^A RNA methylation in the FTO knockdown group was significantly higher than in the vulnerable plaque group and empty load group (*P*<0.05). The levels of total cholesterol, triglyceride, and low-density lipoprotein C were higher at the 12^th^ week than at the 1^st^ week, but the high-density lipoprotein C level was lower at the 12^th^ week than at the 1^st^ week. At the 12^th^ week, the interleukin-7 level was significantly lower in the adeno-associated virus-9 (AVV9)-FTO short hairpin RNA group than in the control and AVV9-green fluorescent protein groups (*P*<0.001).

**Conclusion:**

After successfully establishing a vascular parkinsonism rabbit model, m^6^A RNA methylation can decrease Th cells and vulnerable atherosclerotic plaques.

## INTRODUCTION

In the modern society, with the improvement of living standards, the incidence of cardiovascular diseases is rising rapidly. The epidemiological investigation has showed that the mortality rate of cardiovascular diseases ranks first in all major cities, even in the whole China^[1]^. The main pathological basis of cardiovascular disease is the formation and evolution of atherosclerotic plaque. Current studies have shown that vascular parkinsonism (VP) is the comprehensive mechanism for atherosclerotic plaque, mainly including inflammatory immune response, oxidative stress response, apoptosis, autophagy, vascular remodeling, lymphatic neovascularization, plaque stress, and shear force^[2]^. The inflammatory immune response throughout the formation of VP is the core mechanism. Batista et al.^[3]^ first elucidated the biological role of *N*^^6^^-methyladenosine (m^6^A) modification in T cell-mediated pathogenesis in 2017, and they confirmed that m^6^A ribonucleic acid (RNA) demethylation controlled T cell homeostasis by targeting interleukin (IL)-7/signal transducer and activator of transcription 5 (STAT5)/suppressors of cytokine signalling (SOCS) to inhibit the occurrence of enteritis. It provides a new way for m^6^A RNA demethylation to regulate cellular immunity.

As an important intermediate medium in the immune response, helper T (Th) cells play a role in stabilizing other immune cells. Through self-proliferation, they indirectly activate other types of immune cells to directly act on inflammatory responses^[4]^. It has been reported that IL-7 is the main regulator of T cell homeostasis, and its main function is to promote the adhesion of white fine cells to endothelial cells during inflammation^[5]^. Batista et al.^[3]^ have demonstrated that m^6^A RNA demethylation can control T cell homeostasis by targeting IL-7/STAT5/SOCS, and IL-7 is closely related to homeostasis, proliferation, and differentiation of Th cells. However, whether Th cells undergo m^6^A RNA methylation by themselves has not been clearly studied.

In our previous study, high-fat feeding, balloon damage, and p53 gene transfection techniques have been applied to successfully establish the VP model of atherosclerosis^[6]^. It has been confirmed that statin drugs can achieve partial stabilization of plaque by inhibiting inflammatory factors. Therefore, the purpose of this study was to explore the effect of m^6^A RNA methylation on atherosclerotic vulnerable plaque by knockdown of fat mass and obesity-associated gene (FTO), so as to provide the experimental basis for gene therapy to stabilize VP through the immune mechanism in the future.

## METHODS

### Animal Model

The three-month-old New Zealand white rabbits were purchased from the experimental animal center of Xuzhou Medical University (Xuzhou, China). The experimental design was approved by the laboratory animal ethics committee of Xuzhou Medical University (license number: SYXK (Su) 2015-0029, certificate number: 201904540).

A total of 50 New Zealand white rabbits were randomly divided into pure high-fat group (high-fat feeding), sham operation group (sham operation + high-fat feeding), vulnerable plaque group (operation + high-fat feeding + adenovirus mediated p53 gene [Ad-p53] transfection), empty load group (operation + high-fat feeding + Ad-p53 transfection + adeno-associated virus-9 [AVV9] empty load), and FTO knockdown group (operation + high-fat feeding + Ad-p53 transfection + AVV9-FTO knockdown), with 10 rabbits in each group. The rabbits were weighed and placed in a fixed cage, and they were anesthetized with 1% sodium pentobarbital (10 mg/ml, Sigma Chemical Co., St. Louis, Missouri, United States of America) by intravenously injection through the ear margin. After successful anesthesia, they were fixated on the animal operating table. The upper side of the right femoral artery (the strongest pulsation point) was selected as the surgical site, the hairs were shaved, and the site was disinfected and dissected. Subcutaneous tissues and muscles were separated layer by layer, with a blunt separation of about 3 cm from the femoral artery. The femoral artery was clipped with a hemostatic clip, and a small orifice was punctured with a no. 7 needle. A guiding needle was inserted. The hemostatic clamp and observation guide needle arterial bleeding obvious were loosened and placed into the 0.014-inch thread. After pulled the guided needle, 15-mm diameter balloon catheter (diluted with 1:15 heparin saline infiltration) was placed into the abdominal aorta. Then a balloon catheter with a diameter of 3.5 mm and a length of 15 mm (infiltrated with 1:15 diluted sodium heparin and normal saline) is fed into the abdominal aorta about 10 cm along the guide wire. The distilled water was pushed up until the balloon was filled with eight atmospheric pressure, then the balloon was pulled back to the common iliac artery pressure. We repeatedly did this three times to ensure the abdominal aorta endothelial damage. The balloon catheter was taken out after local wound bleeding. Tie thin lines on both ends of the femoral artery. Penicillin (Huabei Pharmaceutical Co., Ltd., Shijiazhuang, China) was given locally to the muscles to prevent infection.

### Collection of Blood Samples

The blood samples were collected at the 1^st^ week and the 12^th^ week. After all rabbits fasted for 12 hours or more, their fasting blood was extracted through the ear vein with scalp needle for biochemical examination. After centrifugation (1000 ×g) for 20 min, the supernatant serum was collected for further detection.

Detection of the levels of total cholesterol (TC), triglyceride (TG), high-density lipoprotein C (HDL-C), and low-density lipoprotein C (LDL-C)

The levels of TC, TG, LDL-C, and HDL-C in serum were detected by using Erba XL-600 automatic biochemical analyzer and commercially available diagnostic kits (Nanjing Jiangcheng Bioengineering Institute, Nanjing, China) in accordance with the instructions.

### Histopathology

The rabbits were killed by cervical dislocation. The corresponding arteries were taken from the rabbits in the high-fat group and sham group. The plaque tissues in the vulnerable plaque group, empty load group, and FTO knockdown group were taken from the marker. After washed with normal saline, the tissues were frozen at -80°C. After rinsed with normal saline, the vessels were immersed in 10% formalin (Hubei Xingyinhe Chemical Co., Ltd., Wuhan, China) and fixed for at least 24 hours. After routine histological treatment, 5-µm sections were cut and stained with hematoxylin and eosin. Histological analysis was performed under an optical microscope (OLYMPUS BX41, OLYMPUS, Tokyo, Japan) and photographed at 100× magnification.

### Detection of the IL-7 Level

At the end of the 12^th^ week, the corresponding arteries in the five groups were taken to measure IL-7 levels. The IL-7 level was detected by enzyme linked immunosorbent assay (or ELISA) with commercially available diagnostic kit (Nanjing Jiangcheng Bioengineering Institute, Nanjing, China) in accordance with the instructions.

### Detection of Th Cells

Annexin V-fluorescein isothiocyanate/propidium iodide (V-FITC/PI) kit (Takara company, Japan) was used to detect Th cells in each group. The isolated tissues were digested with trypsin. Cells were collected by centrifugation (1000 ×g, 10 min) and washed with pre-cooled phosphate buffer. 1× binding buffer was added to resuspend 100 µl cells. 5-µl Annexin V-FITC and 5-µl PI were added to the cell suspension. After gently mixed and incubated in dark for 15 min at room temperature, the ratio of Th cells in each group was determined by flow cytometry.

### Detection of m^6^A RNA Methylation

Total RNA of Th cells was extracted using a tissue/cell total RNA isolation kit (Tiangen Biotech Co., Ltd., Beijing, China) under the conditions recommended by the manufacturer. Reaction buffer and 5 µl supernatant were added into 96 well plate. The operation was performed according to the instructions of commercially available diagnostic kit (Bradford). The absorbance was detected at 405 nm.

### Detection of FTO Protein Expression

The tissues of each group were lysed on ice with lysis buffer (Beyotime Institute of Biotechnology, Shanghai, China) for 10 min. The supernatant was obtained by centrifugation (1000 ×g) at 4°C for 20 min. After sodium dodecyl sulfate-polyacrylamide gel electrophoresis (BIO-RAD Co., California, United States of America), the protein was transferred to nitrocellulose membrane (Pall Co., New York, United States of America). After being blocked with nonfat dried milk (Sangon Biotech Inc., Shanghai, China), the membrane was incubated with anti-FTO antibody anti-β-actin antibody (Abcam Technology, Cambridge, United Kingdom) overnight at 4°C. The membrane was then incubated with secondary antibody (ZSGB Biotech Co., Ltd., Beijing, China) for one hour at 25°C. Enhanced chemiluminescence solution was added to the darkroom for development, exposure, and photography by gel imager. With β-actin as internal reference, the data were analyzed by QuantityOne image analysis software.

### Statistical Analysis

Statistical analyses were performed by MATLAB 2016b software. The measurement data were expressed as means ± standard deviation. *t*-test and Chi-square test were used for the comparison of differences between two groups. One-way analysis of variance was used for the comparison among multiple groups. *P*<0.05 was considered as statistical difference.

## RESULTS

### Successful Establishment of the Animal Model of VP

During the whole experiment, eight New Zealand rabbits died, including two in the sham group (died from infection or anesthesia), two in the vulnerable plaque group (died from anesthesia), two in the empty load group (died from infection) and two in the FTO knockdown group (died from anesthesia or infection). Finally, 42 rabbits survived.

### Biochemical Indexes

Blood samples were taken at the end of the 1^st^ week and the end of the 12^th^ week to analyze the levels of TG, TC, LDL-C, and HDL-C. At the end of the 12^th^ week, six rabbits were randomly selected from the five groups. There were no statistical significances in the levels of TG, TC, LDL-C, and HDL-C among the five groups (*P*>0.05) (Table 1).

**Table 1:** Levels of TC, TG, HDL-C, and LDL-C in the five groups (mmol/L).

Items	Pure high-fat group	Sham operation group	Vulnerable plaque group	Empty load group	FTO knockdown group
TC					
Week 1	1.295±0.061	1.298±0.057	1.300±0.064	1.306±0.056	1.291±0.062
Week 12	10.24±0.657	10.58±0.924	10.82±0.911	10.68±0.907	10.28±0.807
LDL-C					
Week 1	0.519±0.062	0.525±0.053	0.522±0.067	0.520±0.061	0.531±0.055
Week 12	7.162±0.275	7.684±0.249	7.531±0.285	7.272±0.278	7.472±0.328
HDL-C					
Week 1	0.958±0.145	1.105±0.136	1.079±0.119	1.109±0.124	1.089±0.112
Week 12	0.657±0.093	0.652±0.051	0.650±0.108	0.655±0.103	0.635±0.111
TG					
Week 1	1.151±0.111	1.135±0.327	1.094±0.162	1.097±0.163	1.091±0.153
Week 12	4.604±0.355	4.886±0.221	4.754±0.375	4.734±0.365	4.712±0.402

FTO=fat mass and obesity-associated gene; HDL-C=high-density lipoprotein C; LDL-C=low-density lipoprotein C; TC=total cholesterol; TG=triglyceride

### Histopathological Results of Abdominal Aorta

**Fig. 1:**
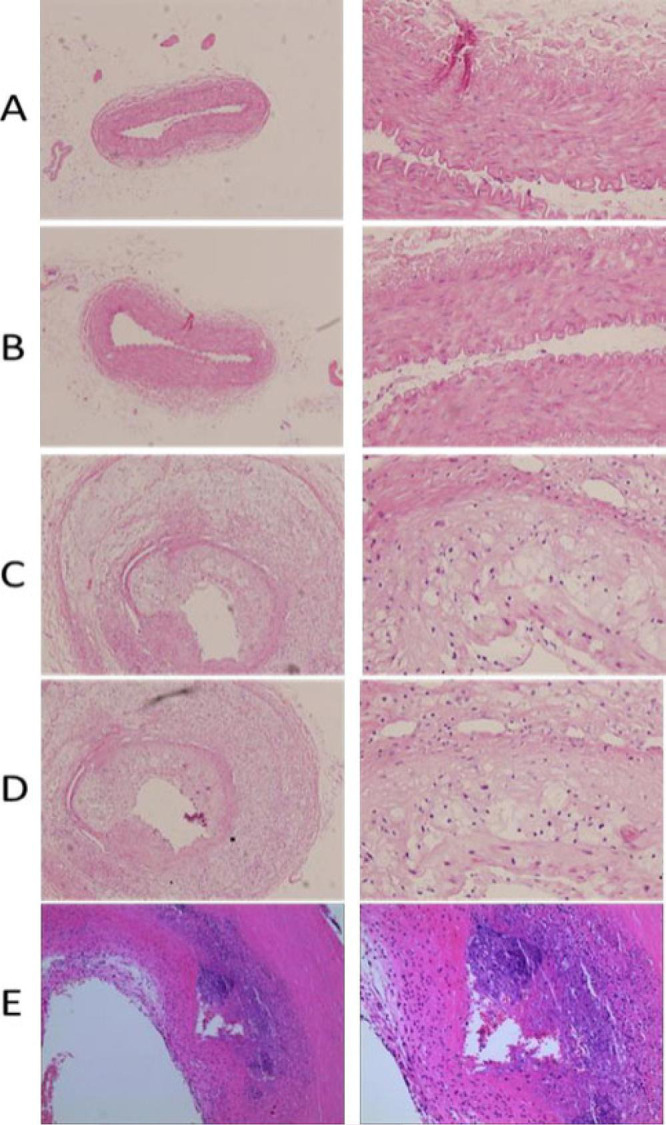
Histopathological changes in the five groups. A, B, C, D, and E represent the pure high-fat group, the sham operation group, the vulnerable plaque group, the empty load group, and the fat mass and obesity-associated gene knockdown group, respectively. From left to right, the magnification was 10× and 40×.

The histopathological changes in the five groups are shown in Figure 1. The inner membrane of cells in the pure high-fat group and the sham group were smooth and uniform, but no foam cells were observed. A large number of foam cells were observed in the vulnerable plaque group, the empty load group, and the FTO knockdown group, with thinner blood vessel walls, exfoliated endothelium, and significantly narrowed lumen. Compared with the vulnerable plaque group and empty load group, platelet aggregation was more obvious in the FTO knockdown group.

### Il-7 Levels

There was no significant difference in the IL-7 level between the pure high-fat group and the sham group (*P*=0.251), and between the vulnerable plaque group and the empty load group (*P*=0.471). The IL-7 levels in the vulnerable plaque group, FTO knockdown group, and empty load group were statistically higher than in the pure high-fat group (*P*<0.01), while that in the FTO knockdown group was statistically higher than in the vulnerable plaque group (*P*<0.01). The results are shown in Table 2.

**Table 2:** IL-7 level in the five groups at the 12^th^ week.

Groups	Pure high-fat group	Sham operation group	Vulnerable plaque group	Empty load group	FTO knockdown group
Survive number	10	8	8	8	8
Average	7.78±1.60	8.72±1.63	12.88±1.98^^*^^	11.41±0.73^^*^^	16.42±1.3^^*^#^

*P*<0.01 vs. pure high-fat group;

# P<0.01 vs. vulnerable plaque group

FTO=fat mass and obesity-associated gene; IL=interleukin

### Western Blot Analysis of FTO Knockdown

**Fig. 2:**
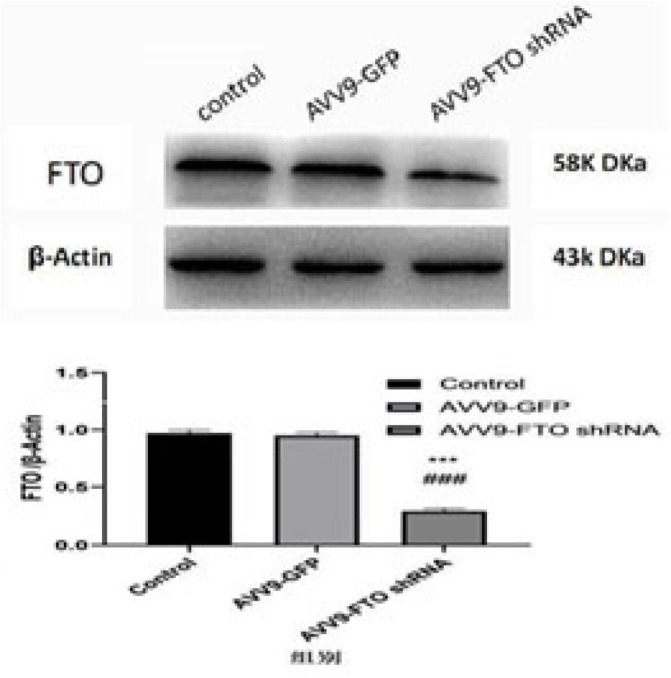
Western blot results of fat mass and obesity-associated gene (FTO) protein expression in the vulnerable plaque group, empty load group, and FTO knockdown group. ***P<0.001 vs. control group; ###P<0.001 vs. AVV9-GFP group. AVV9=adeno-associated virus-9; GFP=green fluorescent protein; shRNA=short hairpin ribonucleic acid.

At the end of the 12^th^ week, three rabbits were taken from the vulnerable plaque group, empty load group, and FTO knockdown group. The vulnerable plaque group was set as control group, the empty load group as AVV9-green fluorescent protein group, and the FTO knockdown group as AVV9-FTO short hairpin RNA group. Figure 2 shows that there was no significant difference in FTO protein expression between the vulnerable plaque group and the empty load group (*P*>0.01). The FTO protein expression level in the FTO knockdown group was significantly lower than those in the vulnerable plaque group and the empty load group (*P*=0.001).

### m^6^A RNA Methylation Results

**Fig. 3:**
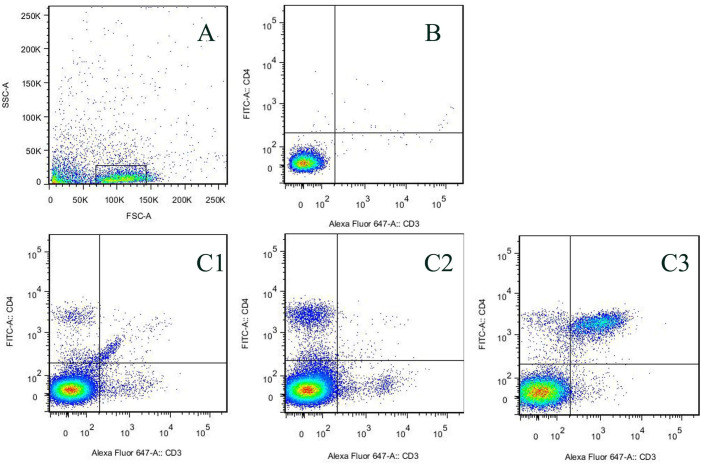
T helper cells in the five groups detected by flow cytometry. CD3=anti-CD3; CD4=anti-CD4; FITC-A=fluorescein isothiocyanate-area; FSC-A=forward scatter-area; SSC-A=side scatter-area.

**Fig. 4:**
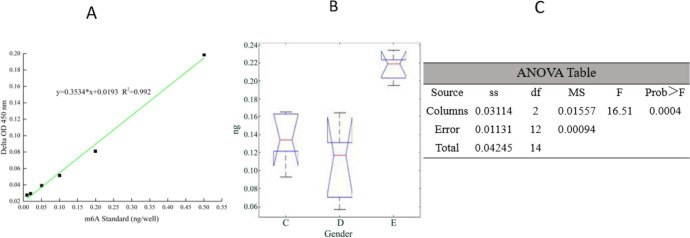
Correlation diagram of N^^6^^-methyladenosine (m^6^A) ribonucleic acid methylation results. ANOVA=analysis of variance. df=degrees of freedom; F=F-ratio; MS=mean square; SS=sum of squares.

The Th cells in the vulnerable plaque group, empty load group, and FTO knockdown group were screened by flow cytometry (Figure 3). After screening, RNA was extracted from the Th cells and m^6^A methylation was measured by colorimetry. The results showed that there was no significant difference between the vulnerable plaque group and empty load group (*P*=0.2198). As shown in Figure 4, the m^6^A methylation in the FTO knockdown group was significantly higher than in the vulnerable plaque group and the empty load group (*P*=0.001). This indicated that after FTO knockdown, m^6^A methylation value in the Th cells was higher than in the non-knockdown group.

## DISCUSSION

The pathological mechanism of atherosclerotic plaque formation is complicated. In this experiment, the VP rabbit model was involved in the balloon from the femoral artery to strain the endothelium, and the inflammatory reaction was formed locally. The atheromatous plaque was successfully formed through high-fat feeding. The stability of the plaque was reduced through local injection of Ad-p53. Finally, VP was formed, which well simulated the whole process of VP formation.

Our results showed that the expression of histone in FTO knockdown was significantly decreased, indicating that FTO knockdown was successful. The methylation level in the Th cells in the FTO knockdown group was significantly higher than that in the vulnerable plaque group and empty load group, indicating that the regulation of m^6^A RNA could affect the methylation level of Th cells. The IL-7 level, an inflammatory marker, and the proportion of Th cells in lymphocytes were also significantly increased in the FTO knockdown group. We considered the following three possibilities. m^6^A RNA increases the IL-7 level, and IL-7 plays a role in the proliferation of Th cells. The proliferating Th cells indirectly activate other types of immune cells, making them directly affect the inflammatory response and indirectly affect VP. Moreover, this is consistent with the results of Batista et al.^[3]^ Methylated Th cells can activate other types of immune cells indirectly through self-proliferation. Furthermore, methylated Th cells limit self-proliferation, contrary to the proliferation effect of IL-7 on Th cells. However, it mainly be the proliferation effect of IL-7 on Th cells.

The research on RNA methylation is at the forefront. At present, the RNA methylation modification has been defined to two types, namely m^6^A and 5-methylcytosine (or m^5^C), with m^6^A as the main mode. m^6^A is the most abundant internal modification in micro RNA, mainly occurring on the consistent moduli of G[G>A]m6AC[U>A>C]^[7-10]^. Although m^6^A was first discovered in the 1970s^[11,12]^, the lack of technology to study RNA modification limits m^6^A research, and the field has not advanced for decades. In 2012, a full transcriptome method for immunoprecipitation of m^6^A RNA was reported, followed by the next generation of sequencing (m^6^A-seq or merip-seq), which detected m^6^A peaks in over 7,000 messenger RNA (mRNA) transcripts and hundreds of long non-coding RNAs in human and mouse cells, many of which were conserved between humans and mice^[13,14]^. The next research has showed that mRNA or non-coding RNA decorated m^6^A played a key role in organization development, stem cell self-renewal and differentiation, heat shock response and circadian rhythm control, and RNA fate and function, such as mRNA stability, splicing, transport, positioning and translation, interaction between proteins and RNA - primary micro RNA processing^[15-24]^ -, tumor stem cell growth, self-renewal and tumorigenesis^[25-28]^, RNA metabolism, including tiny deoxyribonucleic acid/RNA/low nuclear acid RNA biology, processing, and export^[29-31]^.

In 2016, He Chuan published a review on m^6^A RNA in Nature Reviews Genetics, which fully analyzed the mechanism of m^6^A^[32]^. As the most common post-transcriptional modification on eukaryotic mRNA, over 80% of RNA bases are associated with methylation. It was found that m^6^A accelerated mRNA metabolism and translation modification in cell. It plays an important physiological role in cell differentiation, embryo development, and immune response. Li Huabing, Batista, and other Chinese and American co-researchers published a study in Nature in 2017. They first elucidated the biological role of m^6^A modification in the pathogenesis mediated by T cells, confirming that m^6^A mRNA demethylation controls T cell homeostasis by targeting IL-7/STAT5/SOCS pathway, thus inhibiting the occurrence of colitis. A large number of studies have shown that m^6^A plays an extensive and important role in the regulation of mRNA. The selection of m^6^A in this study was based on these studies to explore the effect of m^6^A on VP.

In the past, we treated atherosclerotic plaque more from the perspective of etiology to control the risk factors. As one of the risk factors, immune factors can be controlled by few methods. It is assumed that by regulating the level of relevant factors, local inflammatory responses can be controlled, and the evolution of plaque to VP of atherosclerosis can be inhibited, which will greatly slow down the formation of VP of atherosclerosis and provide a new idea for the treatment of atherosclerosis.

Limitations

In previous studies, our group has successfully established a rabbit VP model, which is based on local transfection of Ad-p53 and high-fat feeding on the basis of arterial endothelial balloon strain. In this study, based on the amount of tissue required for the plaque model (the amount of inflammatory cells in atherosclerotic VP is not large, and it is difficult to extract Th cells), we chose the atherosclerotic VP rabbit that was successful in the previous experiment model. There are currently no METL3 and METL14 knockdown types in this model rabbit. We chose to inject AVV-FTO knockdown to achieve local methylation. According to theory, the effect of overall injection of AVV9-FTO is more obvious, but based on the funding and the weight of the rabbit, this operation is impractical, so we choose local injection of AVV9-FTO to knockdown to achieve the effect of methylation.

## CONCLUSION

In conclusion, our study showed that we successfully established a VP rabbit model. m^6^A RNA methylation can decrease Th cells and vulnerable atherosclerotic plaques.
